# Development and preliminary mechanistic analysis of compound effervescent hepatoprotective granules

**DOI:** 10.3389/fnut.2025.1639561

**Published:** 2025-10-15

**Authors:** Xinyao Liu, Ruru Wang, Yumeng Li, Jing Zhang, Jingjing Li, Shuangping Li, Qingping Ye, Renting Cao, Wen Wang, Wenhui Luo, Shujuan Beng, Xianchun Duan, Can Peng

**Affiliations:** ^1^Department of Pharmacy, The First Affiliated Hospital of Anhui University of Chinese Medicine, Hefei, China; ^2^School of Pharmacy, Anhui University of Chinese Medicine, Hefei, China; ^3^Key Laboratory of Xin'An Medicine, Ministry of Education, Hefei, China; ^4^Xin'an Medical Research Institute, Hefei, China; ^5^Anhui Province Key Laboratory of Chinese Medicinal Formula, Hefei, China

**Keywords:** Traditional Chinese medicine, functional food, effervescent granules, liver injury, pharmacology

## Abstract

**Background:**

The causes of acute liver injury (ALI) are complex and diverse, including alcohol, drugs, infections, and toxic substances, and it has become a major global health issue. Traditional Chinese medicine (TCM), with advantages such as broad-spectrum biological activity, low toxicity, and minimal side effects, has been widely used in drug research and development as well as disease management. Some TCMs have shown significant efficacy in treating ALI: Ganoderma lucidum polysaccharides, monoterpene glycosides from Paeonia lactiflora, glycyrrhizic acid saponins, and flavonoids all exhibit liver-protective effects; however, the protective effects of their compound preparations on liver injury remain unclear.

**Methods:**

The study optimized the water extraction process using orthogonal experiments with AHP-entropy weight scoring. The preparation process was verified by single-factor experiments, Plackeett–Burman and Box–Bohnken tests. The mechanism of action was validated using network pharmacology methods and a CCl4-induced acute liver injury animal model.

**Results:**

The developed extraction and granule formation processes were reliable after validation. The contents of active components in CEHG determined by HPLC were as follows: albiflorin 1.26%, paeoniflorin 5.42%, liquiritin 0.43%, glycyrrhizic acid 1.30%, and ganoderic acid A 0.14%, with batch-to-batch variation coefficients (RSD) of 1.66%, 0.87%, 2.32%, 1.60%, and 4.03%, respectively. The dry extract ratio was 18.23% with an RSD of 2.11%. Network pharmacology revealed that CEHG improved liver injury by regulating the HIF-1, p53, and FoxO signaling pathways. Animal experiments indicated that CEHG granules reduce liver function-related aspartate transaminase (AST), alanine transaminase (ALT), total cholesterol (TC), triglyceride (TG), total bilirubin (TBIL), and LDH levels, decrease the levels of inflammatory factors IL-6, IL-1β, and TNF-α and of oxidative stress-related MDA and ROS, and down-regulated the mRNA and protein levels of P53, Bax, and HIF-1α in rats with liver injury. Meanwhile, CEHG improved liver function-related Total Protein (TP), raised the levels of oxidative stress-related SOD, GSH-Px, and GSH, and up-regulated mRNA and protein expression of Bcl-2.

**Conclusion:**

This study successfully optimized the extraction and granule-formation of CEHG and revealed its hepatoprotective mechanism through network pharmacology and animal studies, providing scientific evidence supporting the further development and clinical use of CEHG.

## 1 Introduction

Chemical liver injury (CLI) is a major cause of acute liver injury, whose incidence is rising yearly (1). CLI can result from improper drug use, long-term alcohol consumption, and exposure to toxic chemicals in the environment (2–4). These factors can directly poison liver cells, induce metabolic disorders, trigger oxidative stress, and activate immunological responses, all of which can damage liver cells (5). This damage can lead to different liver diseases that not only lower the quality of life of patients but can also be life-threatening. Thus, the development of liver-protective drugs or functional foods is of great practical significance.

Traditional Chinese medicine (TCM), with its multi-component and multi-target approach, offers great potential for modern drug development. Many TCMs and their extracts can protect the liver by regulating hepatic metabolic function, combating oxidative stress, inhibiting inflammation, and enhancing immunity (6, 7). Modern pharmacological studies have shown that *Ganoderma lucidum* contains components such as Ganoderma polysaccharides, Ganoderma triterpenoids, and sterols that exert hepatoprotective effects. Their mechanisms include antioxidation, free radical scavenging, anti-inflammation, immunomodulation, antifibrosis, and metabolic regulation (8–11). *Paeonia lactiflora* contains compounds such as monoterpene glycosides, triterpenoids, flavonoids, and tannins. The total glycosides, are the main hepatoprotective components and include active compounds such as paeoniflorin and albiflorin, which protect the liver through antioxidative stress, anti-inflammation, anti-cell death, immunomodulation, improvement of microcirculation, and anti-hepatic fibrosis (12–15). *Glycyrrhiza glabra* is a widely used medicinal plant and a TCM with medicinal and edible values that are extensively applied in the food and pharmaceutical industries. Its main chemical components consist of triterpenoid saponins, flavonoids, and polysaccharides. These components protect the liver through mechanisms such as antioxidation, anti-inflammation, anti-apoptosis, detoxification, and regulation of lipid metabolism (16–19). In our study, the following three herbs are combined: *Paeonia lactiflora*, which nourishes the blood, soothes the liver, alleviates pain, and protects the liver, serves as the sovereign herb; *Ganoderma lucidum*, which improves overall health, acted as the ministerial herb; and *Glycyrrhiza glabra*, which strengthened spleen function and harmonized the formula, functioned as the assistant and guide herb. Together, the combination was predicted to form a health-promoting formula that strengthened the body, eliminated harmful influences, balanced qi and blood, and coordinated liver and spleen functions.

Compared with ordinary formulations, effervescent granules have a pleasant taste and are easy to carry. They are widely used in health products and can greatly improve treatment compliance. This study aimed to optimize extraction and granule formation processes to develop a hepatoprotective health products with significant effects and easy acceptance (20). Systematic pharmacological studies and animal experiments will be conducted to verify their protective effects on liver injury, providing a theoretical basis and practical support for the development of new liver-protective health products.

## 2 Materials and methods

### 2.1 Materials

*Ganoderma lucidum* originated from Jinghe, Jilin. *Paeonia lactiflora* was purchased from Huiqiao Pharmaceutical Co., Ltd., Bozhou. *Glycyrrhiza glabra* was purchased from the Jingwan Chinese Medicine Slices Factory, Bozhou. Anhydrous citric acid of food grade, sodium bicarbonate, maltodextrin, and mannitol were obtained from Zhongchen Biotechnology Co., Ltd., Henan. Edible alcohol was acquired from Hanyong Alcohol Co., Ltd., Henan.

### 2.2 Reagents and standards

Ganoderic acid A, paeoniflorin, and albiflorin (purity ≥98%) were purchased from Chengdu Puesday Biological Technology Co., Ltd. Ammonium glycyrrhizinate (purity ≥98%) was obtained from Chengdu Chroma Biotechnology Co., Ltd. Liquiritin (purity ≥98%) was sourced from Beijing Zhongke Zhijian Biotechnology Co., Ltd. HPLC-grade methanol and acetonitrile were bought from Beijing J&K Scientific Technology Co., Ltd. Chromatographic-grade formic acid was procured from Shanghai Aladdin Biochemical Technologies Co., Ltd. The ROS detection kit was purchased from Shanghai Beibio Biotechnology Co., Ltd. The apoptosis kit, MDA colorimetric assay kit, SOD colorimetric assay kit, GSH colorimetric assay kit, GSH-PX colorimetric assay kit, BCA protein concentration determination kit, AST/GOT colorimetric assay kit, ALT/GPT colorimetric assay kit, LDH colorimetric assay kit, TC colorimetric assay kit, TG colorimetric assay kit, TBIL colorimetric assay kit, and TP colorimetric assay kit were obtained from Elabscience. The IL-1β kit, IL-6 kit, and TNF-α kit were purchased from Quanzhou Ruixin Biotechnology Co., Ltd. P53, Bax, Bcl-2, and HIF-1α antibodies were obtained from Affinity. β-actin, goat anti-mouse IgG, and goat anti-rabbit IgG antibodies were purchased from Zs-BIO. RIPA lysis buffer (strong) and PMSF were obtained from Biosharp. SDS, glycine, Tris, APS, acrylamide, bis-acrylamide, and PBS buffer powder were purchased from Servicebio. All other reagents were obtained from professional reagent companies.

### 2.3 Animals

SPF-grade SD rats (male, 7–8 weeks old, 200 ± 20 g, *n* = 60) were purchased from Liaoning Changsheng Biotechnology Co., Ltd. in Liaoning, China [SCXK (Liao) 2020-0001]. The Experimental Ethics Committee of Anhui University of Chinese Medicine (Hefei, China, AHUCM-rats-2024049) granted approval for all animal experiments.

### 2.4 Extraction and preparation of CEHG

#### 2.4.1 Steps of preparation of the CEHG extract

According to the drug usage standards of the 2020 edition of the Chinese Pharmacopeia, the daily formula dosage of CEHG is as follows: *Ganoderma lucidum* 6 g, *Paeonia lactiflora* 6 g and *Glycyrrhiza glabra* 3 g. Preparation of the extract: Weigh the Chinese herbal slices in proportion, place them in a stainless steel pot, and add water to soak (preliminary experiments show that the water absorption rate of the herbal slices is approximately twice their own weight, so an additional twice the amount of water needs to be added for subsequent extraction). After soaking, heat and decoct the mixture; the filtrate is filtered through 8 layers of medical gauze to obtain the extract. Concentrate the extract in a material-to-liquid ratio of 1:1 using a rotary evaporator, freeze-dry it, then crush it in a mortar and pass through an 80-mesh pharmaceutical sieve to obtain the extract powder.

#### 2.4.2 Optimization of extraction process by orthogonal experiment

An orthogonal experimental design with three factors and three levels was adopted to select the levels of the water addition ratio (A), the number of extraction times (B), and the extraction duration (C). The experimental combinations of these factors were arranged according to the orthogonal table, as detailed in Table 1.

**Table 1:** Orthogonal test factors and levels table.

**Level**	**Factors**		
	**Water addition ratio (A, g/ml)**	**Number of extraction times (B)**	**Extraction duration (C, h)**
1	1:10	1	1
2	1:15	2	2
3	1:20	3	3

#### 2.4.3 Optimization of the extraction process by the combined AHP-entropy weighting method

The critical quality indicators of the Compound Liver-protecting Effervescent Granules, determined by the functions of the product, were identified as the dry extract ratio and the content of albiflorin, paeoniflorin, liquiritin, glycyrrhizic acid and ganoderic acid A. The dry extract ratio was measured by the loss on drying method and the contents of albiflorin, paeoniflorin, liquiritin, glycyrrhizic acid and ganoderic acid A were determined by high-performance liquid chromatography (HPLC). Chromatographic column: caprisil C18-AQ (5 μm, 250 mm × 4.6 mm), mobile phase: 0.1% formic acid water (A) acetonitrile (B); gradient elution: 0–8 min, 90%−86%A; 8–16 min, 86%−75%A; 16–21 min, 75%−74%A; 21–26 min, 74%-68%A; 26–48 min, 68%−42%A; 48–53 min, 42%−12%A; 53–54 min, 12%−90%A; 54–55 min, 90%A; flow rate: 0.8 ml/min; detection wavelength: 254 nm; column temperature: 30 °C; injection volume: 10 μl.

Accurately weigh appropriate amounts of paeoniflorin lactone, paeoniflorin, liquiritin, ammonium glycyrrhizinate, and ganoderic acid A, dissolve them in 80% methanol to prepare reference standard stock solutions with mass concentrations of 1.25, 2.66, 1.38, 1.29, and 0.10 mg/ml respectively. Pipette different volumes of the above reference standard solutions into 5 ml volumetric flasks, dilute to the mark, and obtain a mixed reference standard solution with mass concentrations of 62.50, 532.0, 69.00, 129.0, and 25.00 μg/ml respectively.

Accurately weigh 0.2 g of the main drug into a 50 ml volumetric flask, dilute to the mark with 80% methanol, and weigh the mass. Subject the flask to ultrasonic treatment for 30 min (40 kHz, 200 W), then allow it to cool to room temperature. Replenish the lost mass with 80% methanol, shake well, take 1 ml of the solution, centrifuge it at 12,000 r/min for 10 min, and collect the supernatant to obtain the test sample solution.

According to Table 2, paeoniflorin and albiflorin, act as the main hepatoprotective components with equal importance, both scoring 1 point, but slightly more important than liquiritin and glycyrrhizic acid (both scoring 3 points), and significantly higher than ganoderic acid A and the dry extract ratio (both scoring 5 points); AHP hierarchical analysis, subjective and objective entropy weighting of information were carried out using the SPSSAU online analysis tool (https://spssau.com/indexs.html), in which the combined weights were determined according to formula 1, orthogonal experimental data were standardized according to formula 2. A comprehensive score was calculated by comprehensive weighting according to formula 3, and finally the best process was defined through variance and range hierarchical analysis of AHP.


(1) $$\begin{array}{l} W_{j}\;=\;W_{j}^{S}W_{j}^{O}/\sum_{j=1}^{n}W_{j}^{S}W_{j}^{O} \end{array}$$

(2) $$\begin{array}{l} C_{i,j}\;=\;\left(x_{i,j}-\min\left(x_{i,j}\right)\right)/(\max\;(x_{j})\\ \;-\min\;\left(x_{i,j}\right)) \end{array}$$

(3) $$\begin{array}{l} \text{Comprehensive Score }={\text{ C}}_{\text{i},\text{paeoniflorin}}\times {\text{W}}_{\text{paeoniflorin}}+{\text{C}}_{\text{i},\text{albiflorin}}\\ \;\times {\text{W}}_{\text{albiflorin}}+{\text{C}}_{\text{i},\text{glycyrrhizic acid}}\\ \;\times {\text{W}}_{\text{glycyrrhizic acid}}+{\text{C}}_{\text{i},\text{liquiritin}}\times {\text{W}}_{\text{liquiritin}}\\ \;+{\text{C}}_{\text{i},\text{ganoderic acid A}}\times {\text{W}}_{\text{ganoderic acid A}}\\ \;+{\text{C}}_{\text{i},\text{dry extract ratio}}\times {\text{W}}_{\text{dry extract ratio}} \end{array}$$

**Table 2:** Criteria for AHP construction of judgment matrices.

**Scale**	**Meaning**
1	Indicates that the two factors are equally important.
3	Indicates that one factor is slightly more important than the other.
5	Indicates that one factor is significantly more important than the other.
7	Indicates that one factor is strongly more important than the other.
9	Indicates that one factor is extremely more important than the other.
2, 4, 6, 8	Represent intermediate values between the above judgments.
Reciprocal	If the importance of factor *e* over *f* is α*_*ef*_*, then the importance of factor *f* over *e* is 1/*α_*ef*_*

#### 2.4.4 Precision test

Take the mixed reference standard solution, inject it continuously for 6 times under the chromatographic conditions specified in “Section 2.4.3”, record the peak areas and retention times, and calculate the relative standard deviation (RSD) percentage.

#### 2.4.5 Repeatability test

Take the main drug from the same batch, prepare 6 parallel test sample solutions according to the method specified in “Section 2.4.3”, and perform injection analysis. Record the peak areas and retention times, then calculate the relative standard deviation (RSD) percentage.

#### 2.4.6 Stability test

Accurately pipette the same test sample solution, let it stand at room temperature, and perform injection analysis at 0, 2, 4, 8, 12, 16, and 24 h under the chromatographic conditions specified in “Section 2.4.3”. Record the peak areas and retention times, then calculate the relative standard deviation (RSD) percentage.

#### 2.4.7 Determination of molding parameters for CEHG

The extract powder was prepared according to the optimal process parameters. For the preparation of CEHG, the extract powder was first mixed with a diluent (mannitol: maltodextrin = 1:1) and divided into two equal parts. Anhydrous citric acid was added to one part, and sodium bicarbonate was added to the other. The ethanol solution was used as the binder for wet granulation of each part separately; after drying, the granules were mixed uniformly and sized. The amount of extract was fixed at 10 g, whereas the amounts of diluent and binder and the volume fraction of ethanol were varied, with the molding rate used as an index to optimize the process parameters. Based on preliminary experiments, the amount of diluent, the volume fraction of ethanol, and the amount of binder were selected as optimization factors for the Box–Behnken experimental design, with 3 levels set for each. The response surface analysis was performed using Design-Expert v.13.0 software, including 12 factorial points and 5 center points, resulting in a total of 17 experiments. The design is shown in Table 3.

**Table 3:** Factors and levels of box—Behnken experiment.

**Level**	**(a) Amount of diluent/g**	**(b) Volume fraction of ethanol/%**	**(c) Amount of binder/%**
−1	6	80	10
0	10	85	12
1	14	90	14

#### 2.4.8 Verification of an optimal molding process and quality testing of the pulmonary protective effects of CEHG

After determining the optimal process, the validation experiments were conducted in triplicate. The prepared granules were subjected to quality inspection in accordance with the requirements for the granule agents (0104) specified in Volume III of the 2020 Chinese Pharmacopeia. The tests included measurements of particle size, moisture content, dissolving properties, and angle of repose.

### 2.5 Network pharmacology study of compound liver-protecting effervescent granules

#### 2.5.1 Collection of active components and targets

Literature was reviewed to collect active components of Paeonia lactiflora, Glycyrrhiza uralensis, and Ganoderma lucidum. Their SDF (2D) structure files were downloaded from the PubChem (https://pubchem.ncbi.nlm.nih.gov/) platform and imported into the PharmMapper (https://lilab-ecust.cn/pharmmapper/submitfile.html) database to predict potential targets. Data with a normalized fit score ≥0.6 were selected to obtain the UniProt IDs of the targets for each component. These IDs were consolidated and duplicates were removed. Finally, the IDs were converted to gene names via the UniProt (https://www.uniprot.org/) website (21–23).

#### 2.5.2 Collection of liver injury disease targets

Targets related to liver injury were collected by searching the GeneCards (https://www.genecards.org/) database using the keyword “Liver injury” (24). The exported data were filtered in Excel to select target information with a relevance score of at least twice the median value of the relevance scores.

#### 2.5.3 Intersection of active components and liver injury targets

Online tool Venny 2.1.0 (https://bioinfogp.cnb.csic.es/tools/venny/index.html) was used to find the intersection of active component targets and liver injury target genes, and to create a Venn diagram. These intersectional genes (or “genes from the intersection”), regarded as potential targets for the granules against liver injury, were used for further analysis.

#### 2.5.4 Construction of active component–target–disease network

Cytoscape 3.9.1 was used to construct the target network of active components and visualize the relationships between active components, targets, and diseases in the form of an “Active Component–Target–Disease” network diagram. A network topology analysis was performed, and the network style was set based on node degrees. The importance of nodes in the network was reflected by their degree values: the higher the degree value, the larger the node and the darker its color.

#### 2.5.5 PPI network construction and analysis

The intersecting targets were imported into the STRING (http://string-db.org) platform to construct a PPI network, with the organism set to Homo sapiens and a confidence score >0.7 (25). After removing isolated nodes, the network was imported into Cytoscape 3.9.1. Network topology analysis was performed using the CentiScape 2.2 plugin, and key targets were identified based on Degree, Betweenness Centrality (BC), and Closeness Centrality (CC) thresholds.

#### 2.5.6 GO and KEGG enrichment analyses

The intersecting targets were imported into the DAVID (https://davidbioinformatics.nih.gov/) database for GO and KEGG pathway enrichment analyses, with the species set to Homo sapiens and a significance threshold of *P* < 0.01 (26). The top 20 entries were selected and visualized as bar and bubble charts using the WeBioinformatics (http://www.bioinformatics.com.cn/) platform, and the results were analyzed (27).

#### 2.5.7 Molecular docking

Obtain the SDF files of the 2D structures of the screened core components from the PubChem database, then import the SDF files into Chem3D software for energy optimization and conversion into 3D structures; Download the 3D structures of key targets from the PDB database, perform molecular docking using AutoDock software and related plugins, and plot the results using PyMOL software.

### 2.6 Pharmacological study of CEHG on carbon tetrachloride-induced liver injury in rats

#### 2.6.1 Animal grouping and modeling

After 7 days of adaptive feeding in an SPF-grade environment (temperature 23 °C ± 2 °C, 12 h light-dark cycle), 60 rats were randomly divided into 6 groups (10 rats per group). The doses were determined based on the conversion of the human body surface to the rat body area, with low, medium, and high doses set at 5, 10, and 20-fold the human recommended dose, respectively. Carbon tetrachloride (CCl4) (160 mg/kg·BW, dissolved in olive oil) was administered by gavage. Silymarin served as a positive control drug. The experimental groups were as follows: control group (A), model group (B), low-dose group (2.7 g/kg) (C), medium-dose group (5.4 g/kg) (D), high-dose group (10.8 g/kg) (E), and silymarin group (1 g/kg) (F).

The test groups received the samples by gavage daily, whereas the control group and the model group received distilled water. The rats were weighed twice a week to adjust the dose. On day 30, after 16 h of fasting, the model group and the test groups received a single gavage of CCl4, the control group received olive oil, and the test groups continued their respective treatments until the end of the experiment (with a 4-h interval after the gavage with CCl_4_). Twenty-four hours after CCl4 administration, rats were anesthetized with 20% urethane (0.5 mL/100 g), blood was collected for serum separation, and the rats were euthanized by cervical dislocation before the livers were harvested for further analysis.

#### 2.6.2 Pathological histological examination (HE) of the liver

Pathological examination of liver tissue was performed using HE staining of paraffin sections: Paraffin-embedded liver sections were dewaxed with xylene three times (15 min each), then sequentially hydrated with 100%, 95%, and 80% ethanol (5 min each); rinsed under running water until no alcohol remained and the sections were clean and transparent. Sampless were stained with hematoxylin for 3–5 min, rinsed with water, differentiated in 1% hydrochloric acid alcohol for several seconds, then rinsed with water again; treated with dilute lithium carbonate solution for 30 s to restore blue color, rinsed with water, dehydrated with 80% ethanol, and stained with alcohol-soluble eosin for 20 s. The sections were placed in 95% ethanol I and II for about 10 s each for color adjustment, dehydrated in anhydrous ethanol I and II for 1–2 min each, and finally cleared in xylene I and II for 1–2 min each, mounted, and observed under a microscope.

#### 2.6.3 Determination of biochemical indicators in rat serum

Rat blood was collected and allowed to stand for 30 min to 1 h for coagulation. The blood was then centrifuged at 4,000 rpm for 10 min to separate the serum, and the supernatant was collected for analysis. Commercial kits were used to measure the levels of ALT, AST, TG, TC, LDH, TP, TBIL, TNF-α, IL-1β, and IL-6 in rat serum.

#### 2.6.4 Determination of biochemical indicators in rat liver homogenate

Liver tissue was homogenized using PBS (0.01 M, pH 7.4). The homogenate was centrifuged at 10,000 rpm for 10 min at 4 °C and the supernatant was collected on ice. Commercial kits were used to measure the levels of SOD, MDA, GSH, and GSH-Px in the liver homogenate.

#### 2.6.5 Detection of liver tissue ROS levels by flow cytometry

The tissue was homogenized using the homogenate solution according to the ROS kit instructions. After centrifugation, 200 μl of supernatant was retrieved, mixed with 2 μl of probe, and incubated at 37 °C for 30 min. During incubation, cells were mixed to ensure full probe binding. Part of the supernatant was used for protein content determination. After protein measurement, the sample was filtered through a 200-mesh filter and tested using a flow cytometer. The results were analyzed using NovoExpress software.

#### 2.6.6 Detection of apoptotic cells in liver tissue by TUNEL assay

Liver tissue sections were dewaxed with xylene three times (15 min each), then sequentially hydrated with 100%, 95%, and 80% ethanol (5 min each); and rinsed under running water until clean and transparent. The sections were treated with 20 μg/ml proteinase K (without DNase) at 37 °C for 20 min, then washed with PBS three times. The TUNEL detection solution (50 μl; TdT enzyme + Biotin-dUTP=5 μl + 45 μl) was added to each sample, and incubated at 37 °C for 60 min. After washing with PBS three times, 100 μl of reaction termination solution was added and the samples were incubated at room temperature for 10 min. Following 3 washes with PBS, 100 μl of Streptavidin-HRP working solution (0.5 μl + 99.5 μl) was added and the samples were incubated at room temperature for 30 min. After the final three washes with PBS, color was developed using DAB at room temperature for 8 min and the sections were rinsed three times with PBS, counterstained with hematoxylin, to differentiate and restore blue color. Finally, the sections were dehydrated and cleared, mounted with neutral gum, and observed under a microscope.

#### 2.6.7 Detection of apoptotic cells by flow cytometry

Liver tissue samples were ground on ice, centrifuged at 3,000 rpm for 3 min at 4 °C, and the supernatant was collected. Cells were collected at a density of 1–2 × 10^6^ cells, centrifuged at 1,500 rpm for 3 min, and the supernatant was discarded. The cell suspension was then mixed with 2.5 μl of Annexin V-FITC Reagent and 2.5 μl of PI Reagent (50 μg/ml). After gentle vortexing, the mixture was incubated at room temperature in the dark for 15–20 min. Next, 400 μl of diluted 1 × Annexin V Binding Buffer was added and thoroughly mixed. The sample was filtered through a 200-mesh filter and analyzed using a flow cytometer. The flow cytometry images of the apoptotic cells were analyzed using NovoExpress software.

#### 2.6.8 RT-qPCR detection of P53, Bax, Bcl-2, and HIF-1α mRNA expression

Liver tissue processing: 50–100 mg of liver tissue was ground to powder in liquid nitrogen, and 1 mL of TRIzol was added to lyse the cells. After the addition of 0.2 ml of chloroform, the samples were vortexed for 15 s, and incubated on ice for 5 min; and centrifuged at 12,000 rpm for 10 min at 4 °C.

RNA extraction and cDNA synthesis: the supernatant was collected and the RNA was precipitated with isopropanol. The precipitate was washed with 75% ethanol before resuspending in 20–50 μl of DEPC-treated water. RNA was reverse-transcribed into cDNA using one-step first-strand synthesis.

Gene expression: The reaction system contained 10 μl of Taq SYBR Green qPCR Premix (Universal), 0.4 μl each of forward/reverse primers (10 μm), 3 μl of cDNA, and 6.2 μl of RNase-free water, with a total volume of 20 μl. The cycling conditions were as follows: pre-denaturation at 95 °C for 30 s; 40 cycles (95 °C for 15 s, 60 °C for 30 s). Relative quantification was performed using the 2^−Δ*ΔCt*^ method, and primer sequences are listed in Table 4.

**Table 4:** PCR primer sequences.

**Primer**	**Length (bp)**	**Forward primer 5^′^3^′^)**	**Reverse primer 5^′^3^′^)**
Rat-β-actin	150	CCCATCTATGAGGGTTACGC	TTTAATGTCACGCACGATTTC
Rat-P53	85	GGGAATGGGTTGGTAGTTGC	TTTCACTGTAGGTGCCAGGT
Rat-Bax	85	TCATCCAGGATCGAGCAGAG	TTCTTGGTGGATGCGTCCTG
Rat-Bcl-2	95	GCATGCGACCTCTGTTTGAT	CAGGTATGCACCCAGAGTGA
Rat-HIF-1α	121	CGCAGTGTGGCTACAAGAAA	AGGCTGTGTCGACTGAGAAA

#### 2.6.9 Western blot (WB) analysis of protein expression of p53, Bax, Bcl-2, and HIF-1α

Liver tissue samples of 50–100 mg were homogenized in RIPA lysis buffer (containing 1 mM PMSF) at a 1:100 ratio and incubated on ice for 30 min. Samples were centrifuge at 12,000 rpm for 15 min at 4 °C to collect the supernatant as total protein. Protein concentration was determined using the BCA assay kit according to the instructions. 5 × SDS-PAGE loading buffer was added to protein samples at a 1:4 ratio. Samples were heated in boiling water for 10 min to denature the proteins and then loaded onto a 10% SDS-PAGE gel for electrophoresis for 1 h. The proteins were transferred to a PVDF membrane. The membrane was blocked with 5% skim milk for 2 h, and after discarding the blocking solution, the membranes were washed with TBST. Ther membrane was incubated overnight at 4 °C with primary antibodies diluted as follows: p53 (1:1,000), Bax (1:1,000), Bcl-2 (1:1,000), HIF-1α (1:500), and β-actin (1:1,000). After washing, the secondary antibody (1:10,000) was incubated for 2 h. Proteins were detected using an ECL hypersensitive luminescent reagent kit and the grayscale values of the bands were analyzed with ImageJ software. The relative protein expression levels were determined based on by the grayscale densitometric values of β-actin bands.

### 2.7 Statistical analysis

The experimental data were analyzed using GraphPad Prism v.9.4.1. Data are presented as mean ± standard deviation (*x* ± *s*). For comparisons involving multiple groups, one-way analysis of variance (ANOVA) was used. If homogeneity of variances was confirmed, LSD tests were applied for pairwise comparisons. If the variances were heterogeneous, Dunnett-*t* tests were used instead. A *P*-value < 0.05 was considered statistically significant.

## 3 Results

### 3.1 Optimization of the extraction process based on orthogonal experiments and the AHP-entropy method

#### 3.1.1 Optimization of the extraction process

**Figure 1:**
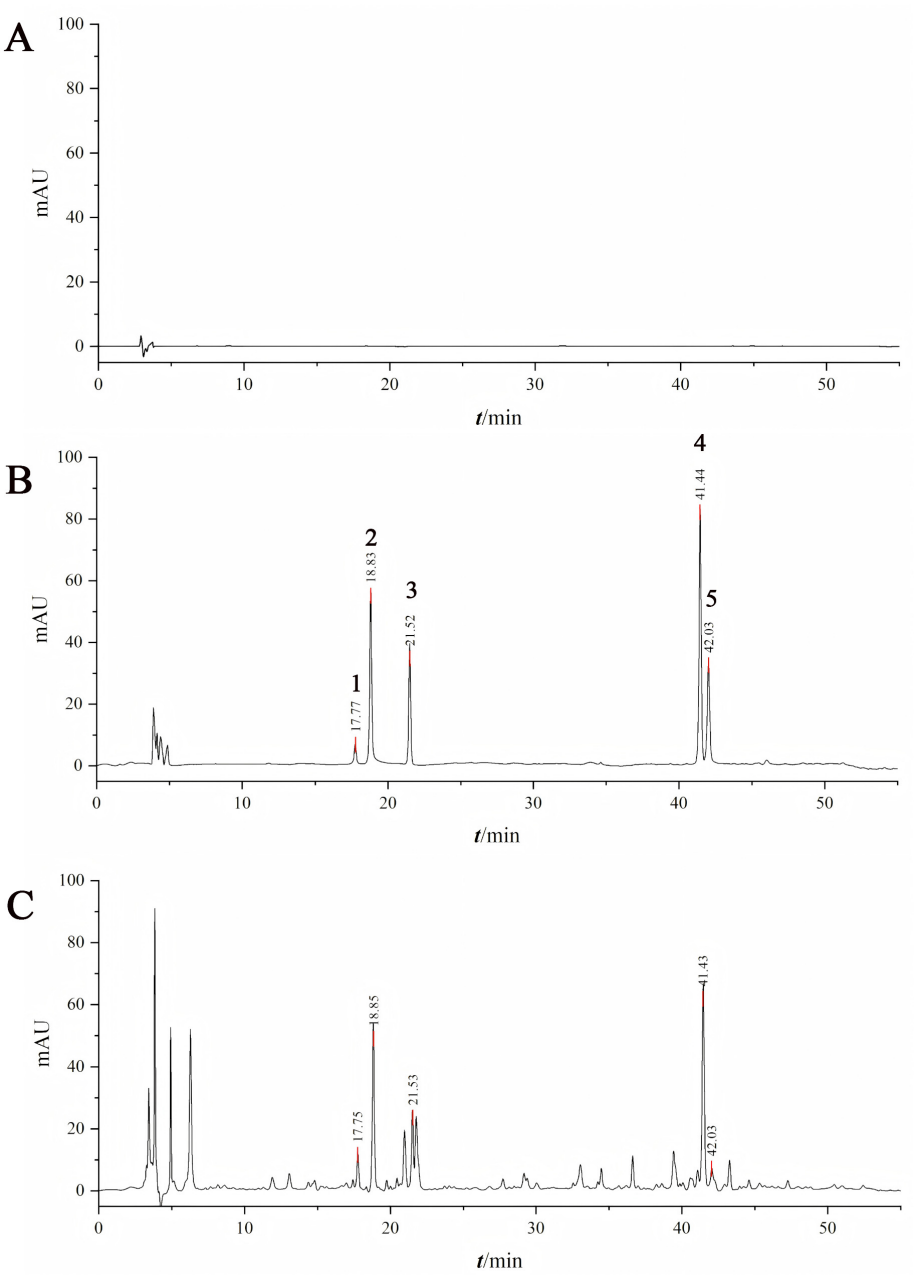
Determination of active ingredient content by HPLC. [**A**: 80% methanol; **B**: Mixed reference standards (1: paeoniflorin, 2: paeonolide, 3: liquiritin, 4: ammonium glycyrrhizinate, 5: ganoderic acid A); **C**: Test sample].

The dry extract ratio and the contents of albiflorin, paeoniflorin, liquiritin, glycyrrhizic acid, and ganoderic acid A in the extract were determined, with the results presented in Table 5 and Figure 1.

**Table 5:** Orthogonal experiment results.

**No**.	**dry extract ratio/%**	**Albiflorin/%**	**Paeoniflorin/%**	**Liquiritin/%**	**Glycyrrhizic acid/%**	**Ganoderic acid A/%**
1	13.93	1.35	5.19	0.42	1.28	0.09
2	21.62	0.86	4.89	0.47	0.93	0.08
3	22.74	1.11	5.02	0.44	1.19	0.09
4	16.32	0.95	4.75	0.45	1.04	0.09
5	20.39	1.11	5.07	0.35	0.92	0.08
6	21.83	0.99	5.22	0.38	0.89	0.05
7	18.61	1.20	5.30	0.38	1.36	0.14
8	17.88	1.09	5.42	0.35	1.21	0.12
9	22.95	0.90	4.76	0.44	1.39	0.16

The subjective weight coefficients based on AHP, the objective weight coefficients based on entropy, and the combined weight coefficients for the ratio of paeoniflorin, albiflorin, glycyrrhizic acid, liquiritin, ganoderic acid A and dry extract are shown in Table 6.

**Table 6:** Weight coefficients.

**Indicator**	**AHP-based subjective weight coefficients**	**entropy-based objective weight coefficients**	**Combined weight coefficients**
Albiflorin	31.85%	17.17%	33.23%
Paeoniflorin	31.85%	9.64%	18.66%
Liquiritin	12.91%	36.01%	28.26%
Glycyrrhizic acid	12.91%	17.18%	13.48%
Ganoderic acid A	5.24%	10.33%	3.29%
Dry extract ratio	5.24%	9.68%	3.08%

After standardization of orthogonal experimental data, the significance of the influence of factors on the results was determined through a range and variance analysis. The intuitive analysis of the R range showed that the primary and secondary factors that affected the comprehensive extraction score were A > C > B (the solid-liquid ratio had the greatest influence, followed by the extraction time, and finally the extraction times). As shown in Tables 7A–C all had significant effects on the complete score (*P* < 0.05). The optimal scheme was A3B1C1 (water addition ratio 1:20, extracted once, 1 h each time). The results of the range analysis and variance analysis are shown in Tables 7, 8, respectively.

**Table 7:** Comprehensive scoring results of the orthogonal test table.

**No**.	**Factors**			**Comprehensive score**
	**A**	**B**	**C**	
1	1:10	1	1	71.59
2	1:10	2	3	26.21
3	1:10	3	2	54.18
4	1:15	1	3	25.15
5	1:15	2	2	25.62
6	1:15	3	1	38.90
7	1:20	1	2	74.45
8	1:20	2	1	63.52
9	1:20	3	3	46.76
K1	151.980	171.190	174.010	
K2	89.670	115.350	154.250	
K3	184.730	139.840	98.120	
k1	50.660	57.063	58.003	
k2	29.890	38.450	51.417	
k3	61.577	46.613	32.707	
R	31.687	18.613	25.297	A>C>B

**Table 8:** ANOVA of orthogonal experiment.

**Source of variance**	**Sum of squares**	**Degrees of freedom**	**Mean square**	***F* value**	**Significance**
A	1554.611	2	777.306	443.800	0.002
B	522.299	2	261.149	149.102	0.007
C	1033.370	2	516.685	294.999	0.003
Error	3.503	2	1.751		

#### 3.1.2 Investigation of precision, repeatability and stability

For the five components (paeoniflorin lactone, paeoniflorin, liquiritin, ammonium glycyrrhizinate, and ganoderic acid A), in the precision investigation, the peak area RSD% values were all in the range of 0.30%−1.63% (all < 2%), and the retention time RSD% values were all in the range of 0.01%−0.09% (all < 0.10%), indicating good precision of the instrument; in the repeatability investigation, the peak area RSD% values were all in the range of 0.24%−1.24% (all < 2%), and the retention time RSD% values were all in the range of 0.03%−0.11% (all < 0.20%), indicating good repeatability of the method; in the stability investigation, within 24 h, the peak area RSD% values were all in the range of 0.67%−1.71% (all < 2%), and the retention time RSD% values were all in the range of 0.02%−0.30% (all < 0.30%), indicating good stability of the test sample within 24 h.

#### 3.1.3 Validation of the extraction process

The results of the validation testing are presented in Table 9. Three batches of parallel tests showed similar index component content and dry extract rates, with RSD values below 5%. This indicated that the extraction process was stable and feasible, with excellent reproducibility.

**Table 9:** Validation experiment results.

**No**.	**Albiflorin/%**	**Paeoniflorin/%**	**Liquiritin/%**	**Glycyrrhizic acid/%**	**Ganoderic acid A/%**	**Dry extract ratio/%**
1	1.25	5.44	0.42	1.28	0.14	18.63
2	1.28	5.37	0.44	1.31	0.15	17.86
3	1.24	5.46	0.43	1.32	0.14	18.21
Mean	1.26	5.42	0.43	1.30	0.14	18.23
RSD%	1.66	0.87	2.32	1.60	4.03	2.11

#### 3.1.4 Optimization of the molding process

The results and analysis of the Box–Behnken experiment are shown in Tables 10, 11. The Box–Behnken experiment yielded a second order polynomial regression equation for the molding rate *Y* = 84.03 – 0.94*a* – 10.26*b* + 1.83*c* – 1.33*ab* + 0.098*ac* + 2.13*bc* – 1.03*a*^2^ – 0.17*b*^2^ – 1.85*c*^2^. As shown in the table below, the model's *F* value was 67.36 and *P* < 0.05, indicating that the model terms were statistically significant. The lack of fit item had a *P*-value > 0.05, indicating a good fit of the regression equation. R^2^ = 0.9886 and Radj^2^ = 0.9739, which indicated that the model had a good fit. Independent variables effectively explained the changes in the response variable. The similar R^2^ and Radj^2^ values suggested that the independent variables introduced were valid. Based on the F value, the significant effects on the formation rate were in the order of *b* > *c* > *a*. In the regression model, the linear terms *b* and *c*, the quadratic term *c*^2^, and the interaction term *bc* were significant, whereas the linear term *a*, the quadratic terms *a*^2^ and *b*^2^, and the interaction terms *ab* and *ac* were not significant. The optimized forming process parameters were as follows: 11 parts of diluent, 12% binder, and 80% ethanol volume fraction.

**Table 10:** Factors and levels table of box—Behnken experimental design.

**No**.	**(a) Amount of diluent/g**	**(b) Volume fraction of ethanol/%**	**(c) Amount of binder/%**	**Molding rate/%**
1	14	80	12	94.23
2	10	80	10	91.67
3	10	85	12	83.96
4	10	90	10	67.52
5	6	85	14	83.67
6	10	85	12	85.07
7	6	85	10	81.40
8	14	85	10	78.44
9	10	90	14	76.62
10	6	90	12	74.09
11	14	85	14	81.10
12	10	85	12	84.87
13	14	90	12	70.45
14	10	80	14	92.27
15	6	80	12	92.56
16	10	85	12	84.14
17	10	85	12	82.12

**Table 11:** Analysis of variance of molding rate.

**Source of variance**	**Sum of squares**	**Degrees of freedom**	**Mean square**	**F-value**	***P*-value**	**Significance**
Model	920.66	9	102.30	67.36	< 0.0001	Significant
A	7.03	1	7.03	4.63	0.0684	
B	841.53	1	841.53	554.11	< 0.0001	^*^
C	26.75	1	26.75	17.62	0.0040	^*^
AB	7.05	1	7.05	4.64	0.0682	
AC	0.0380	1	0.0380	0.0250	0.8787	
BC	18.06	1	18.06	11.89	0.0107	^*^
A^2^	4.50	1	4.50	2.96	0.1290	
B^2^	0.1160	1	0.1160	0.0764	0.7902	
C^2^	14.35	1	14.35	9.45	0.0180	^*^
Residuals	10.63	7	1.52			
Lack of fit	5.18	3	1.73	1.27	0.3985	
Pure error	5.45	4	1.36			
Total error	931.29	16				

^*^P < 0.05, which means the result is significant.

#### 3.1.5 Validation and testing of the optimal molding process of granule quality

Three tests showed a particle formation rate of 95.42%, 96.33%, and 94.76%. The moisture of the CEHG was 5.44%, below 8%. The dissolving time was within 5 min, meeting the Chinese Pharmacopeia requirements. With a resting angle of < 40°, the flow ability of the particles satisfied basic production needs.

### 3.2 Results of the network pharmacology study on CEHG

**Figure 2:**
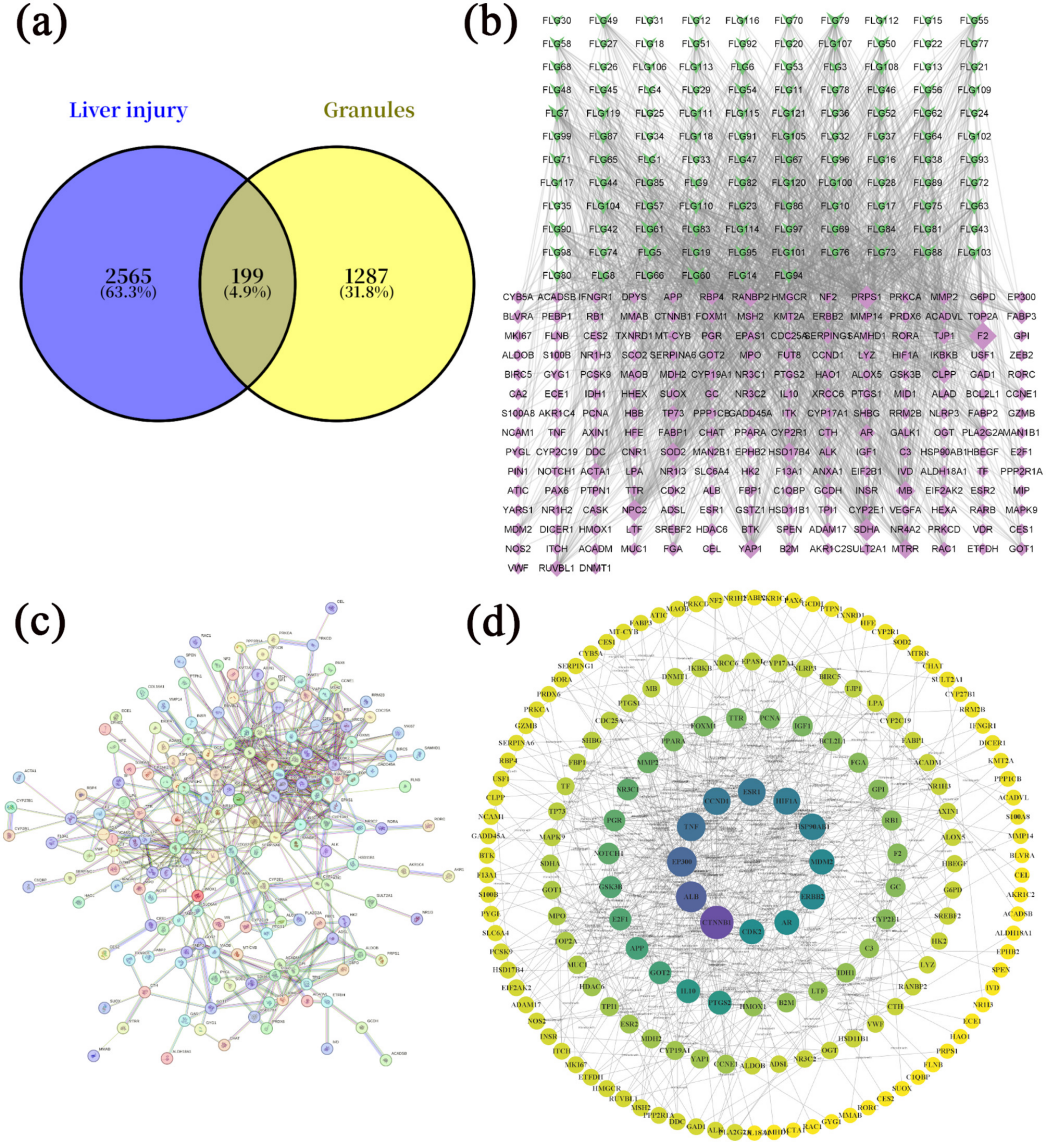
Network pharmacology study of compound liver-protecting effervescent granules (CEHG). **(a)** Intersection of active ingredients and target genes of liver injury; **(b)** network analysis of active ingredients-targets-diseases; **(c)** PPI network analysis an of potential targets for CEHG for the treatment of liver injury; **(d)** core targets map showing results of PPI network analysis of potential targets for CEHG.

**Figure 3:**
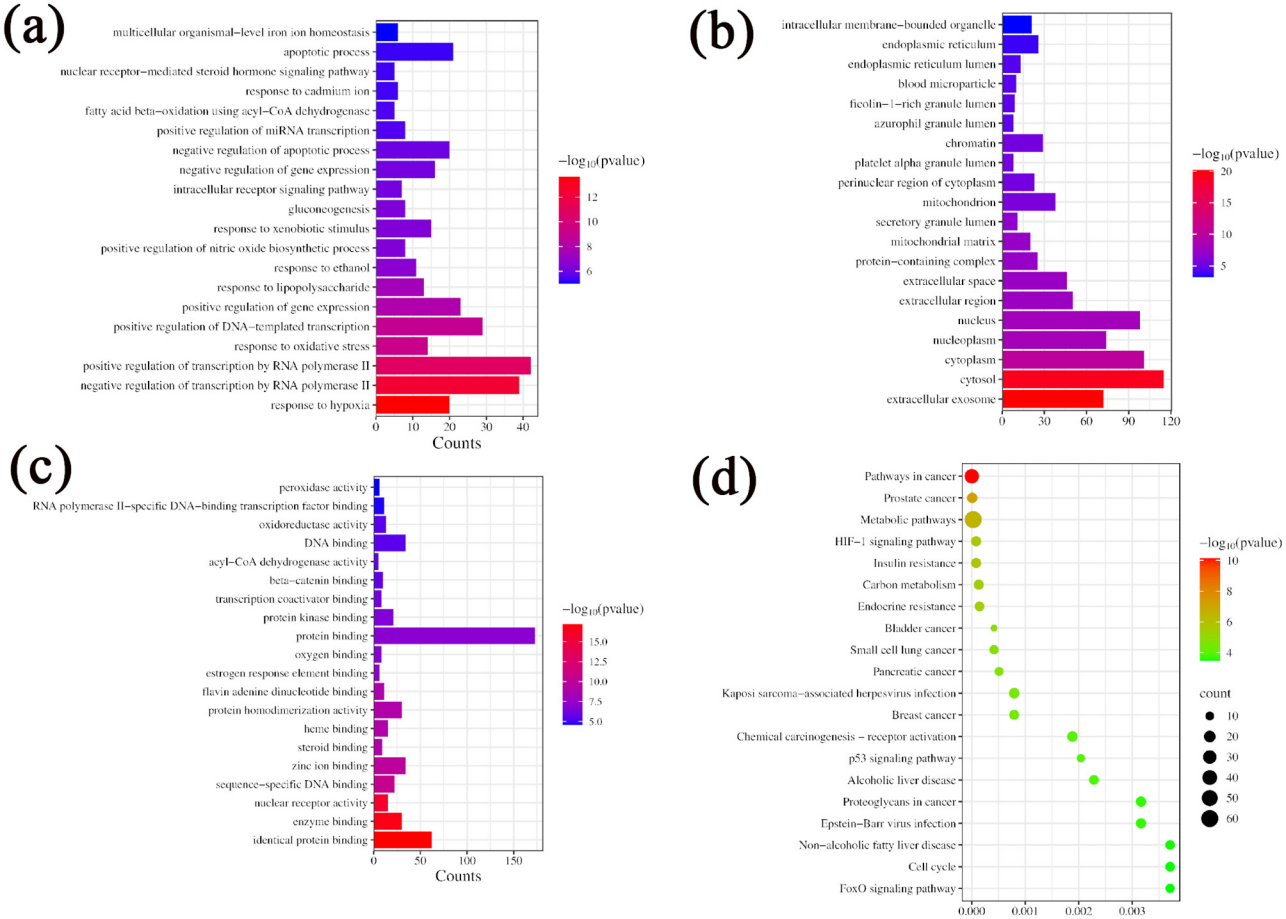
GO and KEGG enrichment analysis. **(a)** GO function enrichment map BP; **(b)** GO function enrichment map CC; **(c)** GO function enrichment map MF; **(d)** KEGG Enrichment Analysis.

**Figure 4:**
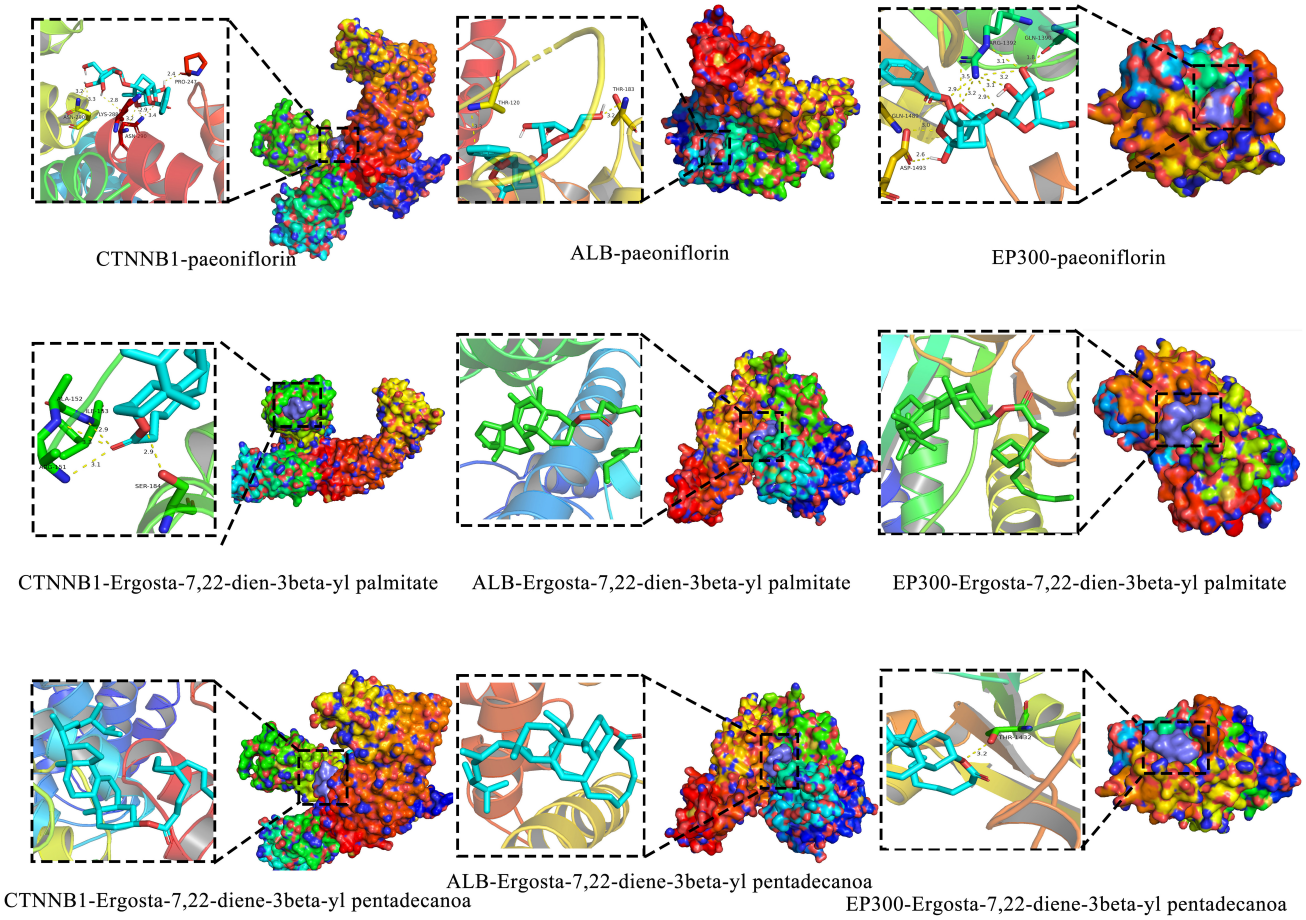
Molecular docking.

A total of 121 bioactive components were identified. After importing into the PharmMapper database for summarization, screening, and deduplication, 1,486 potential targets were identified. Through the GeneCards database, 2,764 targets related to “liver injury” were retrieved. The intersection of the active component target and the liver injury disease target, displayed by a Venny diagram, showed 199 overlapping targets (Figure 2a). The active component-target-disease network diagram revealed key components such as palmitic acid ergosta-7,22-diene-3β-ol ester, pentadecanoic acid ergosta-7,22-diene-3β-ol ester, and paeoniflorin, as well as key targets, including F2, PRPS1 and SDHA (Figure 2b). Based on Degree Centrality (DC), Betweenness Centrality (BC), and Closeness Centrality (CC) thresholds, the analysis of the PPI network identified key nodes such as CTNNB1, ALB, and EP300 (Figures 2c, d). GO enrichment analysis yielded 653 entries (*P* < 0.01), including 445 in BP, 73 in CC, and 135 in MF, with the top 20 entries selected (Figures 3a–c). KEGG pathway enrichment analysis identified 114 signaling pathways (*P* < 0.01), and the top 20 were visualized, which involved signaling pathways such as HIF-1, p53, and FoxO (Figure 3d). Molecular docking was performed between three key components (paeoniflorin, ergosta-7,22-dien-3β-yl palmitate, and ergosta-7,22-dien-3β-yl pentadecanoate) and three key target proteins (ALB, EP300, and CTNNB1). The binding energy of each drug molecule to the corresponding protein was < -5 kJ·mol^−1^, indicating that relatively stable binding can be formed between the drug molecules and the proteins. These drug molecules have the potential to bind to the target proteins, possibly regulate their functions, and thereby exert therapeutic effects on diseases. The molecular docking results are shown in Figure 4.

### 3.3 Pharmacological study of CEHG in the CCl4-induced acute liver injury rat model

#### 3.3.1 Pathological changes induced by CEHG in rats with acute liver injury

**Figure 5:**
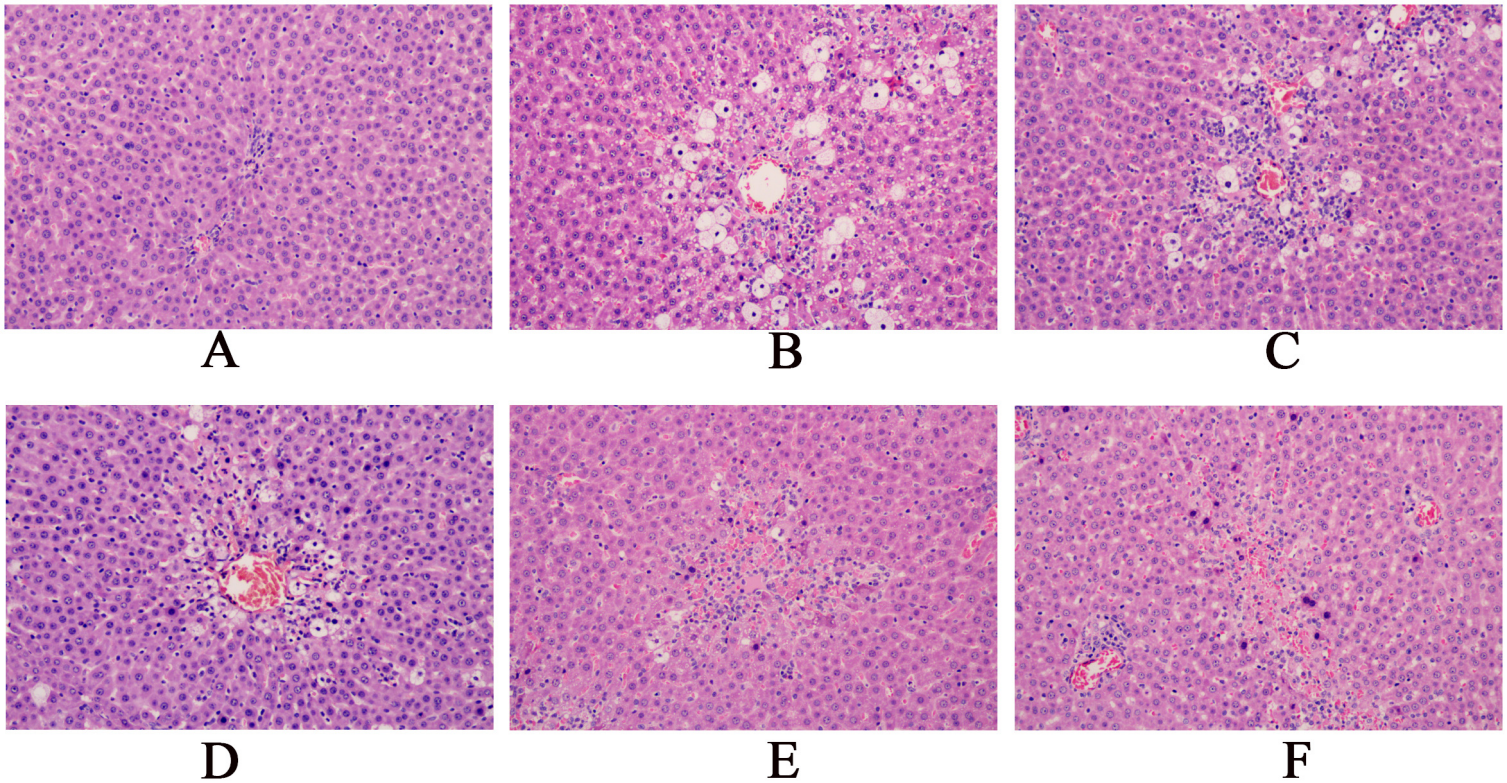
Pathological sections of the rat liver (magnification, x200).(**A**: Blank group; **B**: Model group; **C**: Compound Liver-Protecting Effervescent Granules low-dose group; **D**: Compound Liver-Protecting Effervescent Granules medium-dose group; **E**: Compound Liver-Protecting Effervescent Granules high-dose group; **F**: Silybin group).

HE staining showed that in the control group, rat liver cells were normal in size and shape, with large round nuclei and a clear structure. The hepatocyte cords were arranged radially around the central vein. In the model group of CCl4-induced acute liver injury, significant necrosis, steatosis, inflammatory infiltration, and hemorrhage was observed in liver tissue. As the dose of the granules increased, the liver damage eased and the morphology of the liver improved visibly. Silybin, a reference drug, also improved CCl4-induced liver injury, nearly restoring the tissue to its normal state (Figure 5).

#### 3.3.2 Changes in AST, ALT, TC, TG, TP, TBIL, LDH, IL-6, IL-1β and TNF-α levels in rat serum

**Figure 6:**
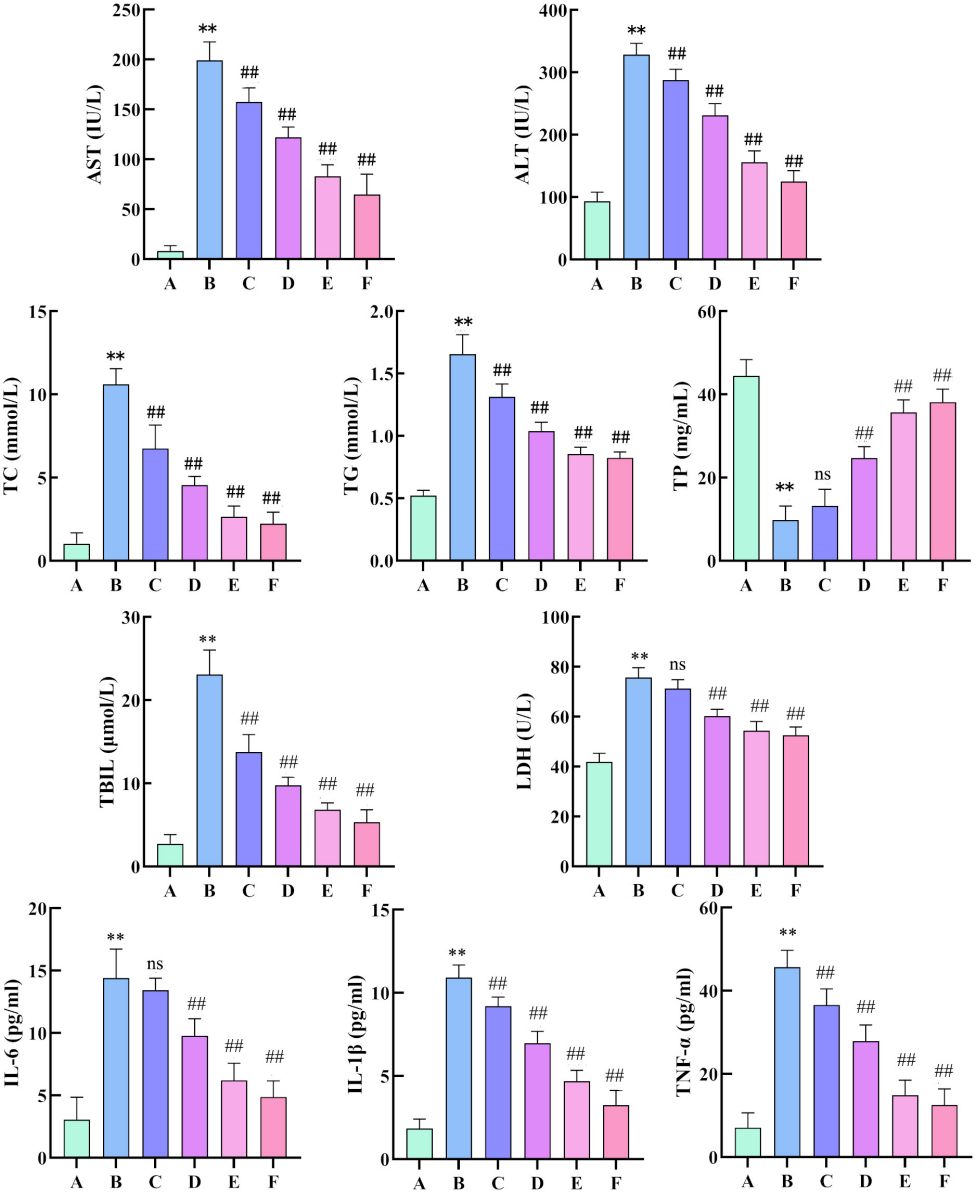
Changes in AST, ALT, TC, TG, TP, TBIL, LDH, IL-6, IL-1β and TNF-α levels in the serum of rats. (*n* = 8, compared with the control group, **P < 0.01, compared with the model group, ^##^*P* < 0.01). (**A**: Blank group; **B**: Model group; **C**: Compound Liver-Protecting Effervescent Granules low-dose group; **D**: Compound Liver-Protecting Effervescent Granules medium-dose group; **E**: Compound Liver-Protecting Effervescent Granules high-dose group; **F**: Silybin group).

Compared with the control group, the model group showed significant increases in ALT and AST, indicating the successful establishment of liver injury models. Compared with the model group, the low, medium, and high dose groups had notable reductions in ALT and AST levels, demonstrating that the effervescent protective granules of compound liver could improve liver function indicators in rats with liver injury in a dose-dependent manner. The positive drug group also had much lower ALT and AST levels than the model group. Compared with the control group, the model group presented higher levels of TC, TG, TBIL, and LDH and significantly lower levels of TP. These changes confirmed the successful establishment of the liver injury model, reflecting lipid metabolic disorders, impaired hepatic synthetic function, and abnormal bilirubin metabolism due to liver injury. Compared with the model group, except for the low-dose group, which showed no significant changes in TP and LDH, all other dosing groups exhibited significantly reduced levels of TC, TG, TBIL, and LDH and significantly increased TP levels. After treatment, TC, TG, TBIL and LDH decreased with increasing doses, whereas TP increased with higher doses. Compared with the control group, the model group showed significantly increased levels of IL-6, IL-1β, and TNF-α. Compared with the model group, all treatment groups, except the low-dose group with non-significant changes in IL-6 levels, exhibited significantly reduced levels of IL-6, IL-1β, and TNF-α. Overall, the liver-protecting effervescent granules of the compound suppressed the release of IL-6, IL-1β, and TNF-α, thus alleviating the inflammatory response (Figure 6).

#### 3.3.3 Changes in the contents of SOD, MDA, GSH, GSH-Px and ROS in rat liver tissue

**Figure 7:**
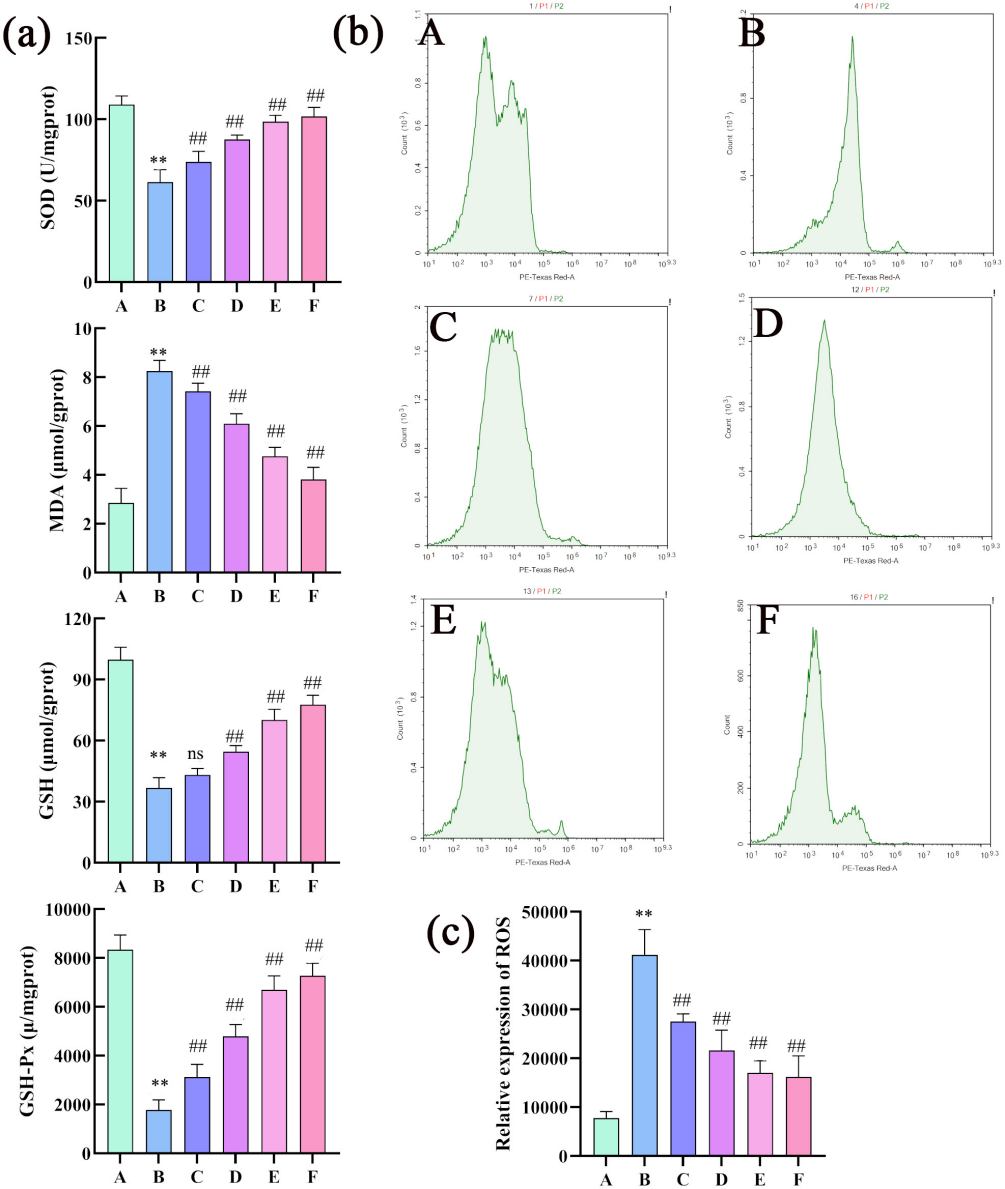
Changes in the SOD, MDA, GSH, GSH-Px, and ROS levels in rat liver tissue (*n* = 8; compared with the control group, **P < 0.01; compared with the model group, ^##^*P* < 0.01; ns indicates that comparisons were not significant. **(a)** Changes in the SOD, MDA, GSH and GSH-Px levels in rat liver tissues (*n* = 8; compared with the control group, **P < 0.01; compared with the model group, ^##^*P* < 0.01; ns indicates not significant); **(b)** changes in ROS levels in rat liver tissues; **(c)** quantitative analysis of ROS (*n* =3 compared with the control group; **P < 0.01, compared with the model group, ^##^*P* < 0.01; ns indicates not significant). Changes in the SOD, MDA, GSH, GSH-Px, and ROS levels in rat liver tissue (*n* = 8); compared with the control group, **P < 0.01; compared with the model group, ^##^*P* < 0.01; ns indicates that comparisons were not significant. **(a)** Changes in the SOD, MDA, GSH and GSH-Px levels in rat liver tissues (*n* = 8; compared with the control group, **P < 0.01; compared with the model group, ^##^*P* < 0.01; ns indicates not significant); **(b)** changes in ROS levels in rat liver tissues; **(c)** quantitative analysis of ROS (n = 3 compared with the control group; **P < 0.01, compared with the model group, ^##^*P* < 0.01; ns indicates not significant).

Compared with the control group, the model group showed significantly reduced SOD and GSH-Px activity, increased MDA levels, and decreased GSH levels. This indicates a weakening of hepatic antioxidant capacity, increased oxidative stress, and severe damage to stem cell membranes in the liver injury model. Compared with the model group, all treatment groups (except for GSH content without significant difference) showed significantly increased SOD and GSH-Px activity and significantly decreased MDA levels and increased GSH levels. Thus, CEHG can protect the liver by enhancing antioxidant capacity and reducing oxidative stress (Figure 7a). Compared with the control group, the model group showed a significant increase in ROS levels in rat liver tissue. Compared with the model group, all treatment groups had markedly lower ROS levels. This indicated that the liver-protecting effervescent granules of CEHG reduced ROS in liver tissue from rats with chemical-induced liver injury, thus decreasing damage due to oxidative stress (Figures 7b, c).

#### 3.3.4 Analysis of cell apoptosis in rat liver tissue

**Figure 8:**
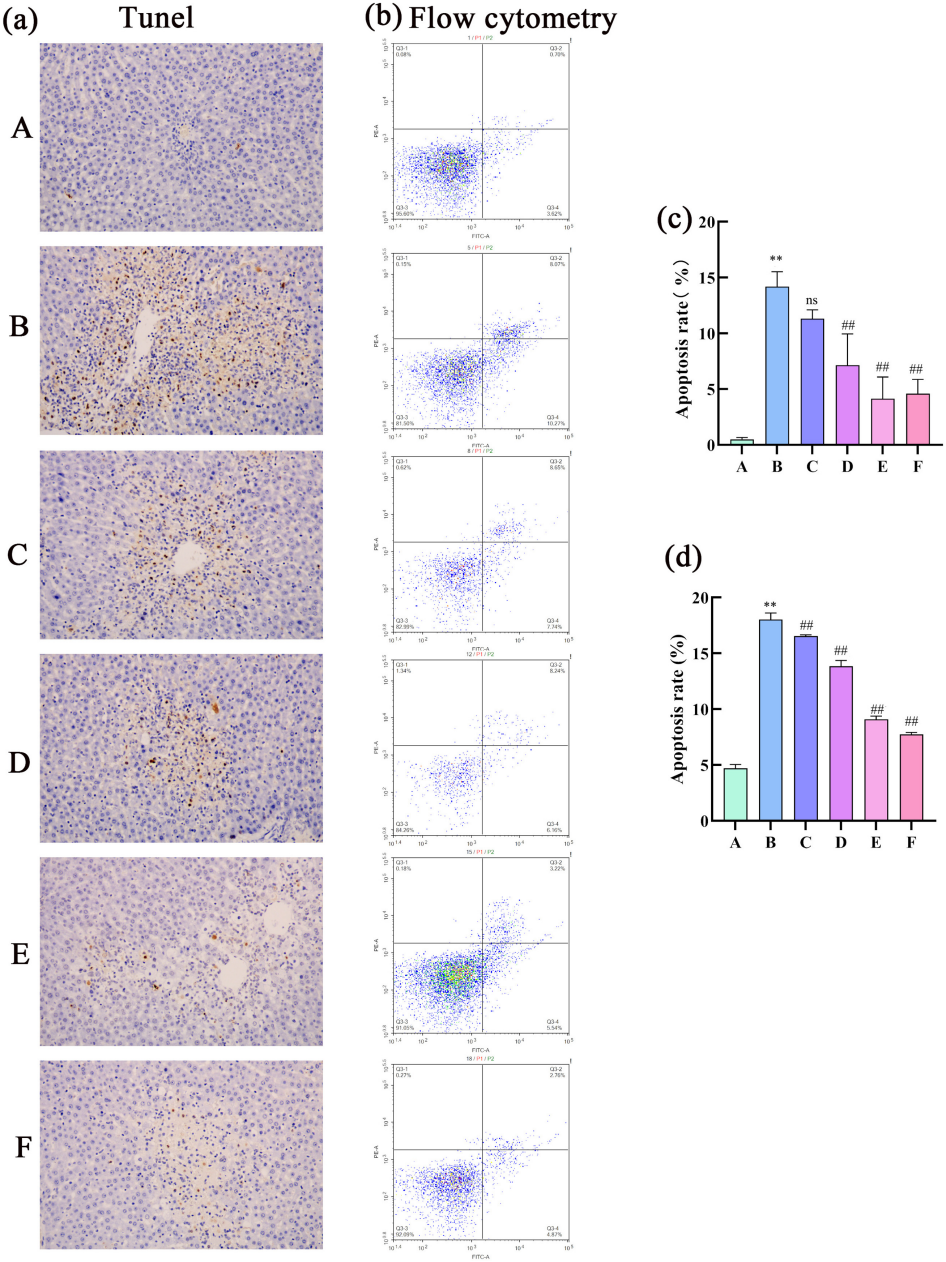
Analysis of cell apoptosis in rat liver tissue. **(a)** TUNEL assay; **(b)** flow cytometry detection of apoptotic cells; **(c)** TUNEL quantification of cell apoptosis (*n* = 3; compared with the control group, **P < 0.01; compared with the model group, ^##^*P* < 0.01; ns indicates not significant); **(d)** Flow cytometry analysis of apoptotic cells (*n* = 3; compared with the control group, **P < 0.01; compared with the model group, ^##^*P* < 0.01).

The results of the TUNEL assay revealed a significantly higher positivity rate in the hepatocytes of the model group than in the control group, indicative of intensified apoptosis. Compared with the model group, the low dose group showed a decrease in positivity rate, albeit not significant, whereas other medicated groups presented markedly lower positivity rates, signaling a substantial reduction in hepatocyte apoptosis. Flow cytometry apoptosis images were analyzed using NovoExpress software as follows: quadrant 1: necrotic cells; quadrant 2: late apoptotic cells; quadrant 3: normal cells; quadrant 4: early apoptotic cells. The combined percentage of cells in quadrants 2 and 4 (late and early apoptotic cells) was calculated. The flow cytometry results showed a higher rate of apoptosis in the model group than in the control group (Figure 8). In contrast, the apoptosis rate was significantly lower in the treatment groups than in the model group, which was consistent with the findings of the TUNEL assay. Overall, these results demonstrate that CEHG can inhibit hepatocyte apoptosis in rats with liver injury, fulfilling their liver-protecting function.

#### 3.3.5 Effects of CEHG on mRNA and protein expression of P53, Bax, Bcl-2, and HIF-1α

The results of RT-qPCR showed that, compared with the control group, the model group had a significant higher mRNA expression of P53, Bax, and HIF-1α, and a lower expression of Bcl-2 mRNA. Compared with the model group, the treatment groups had a significant decrease in P53, Bax, and HIF-1α mRNA levels and increased Bcl-2 mRNA levels.

**Figure 9:**
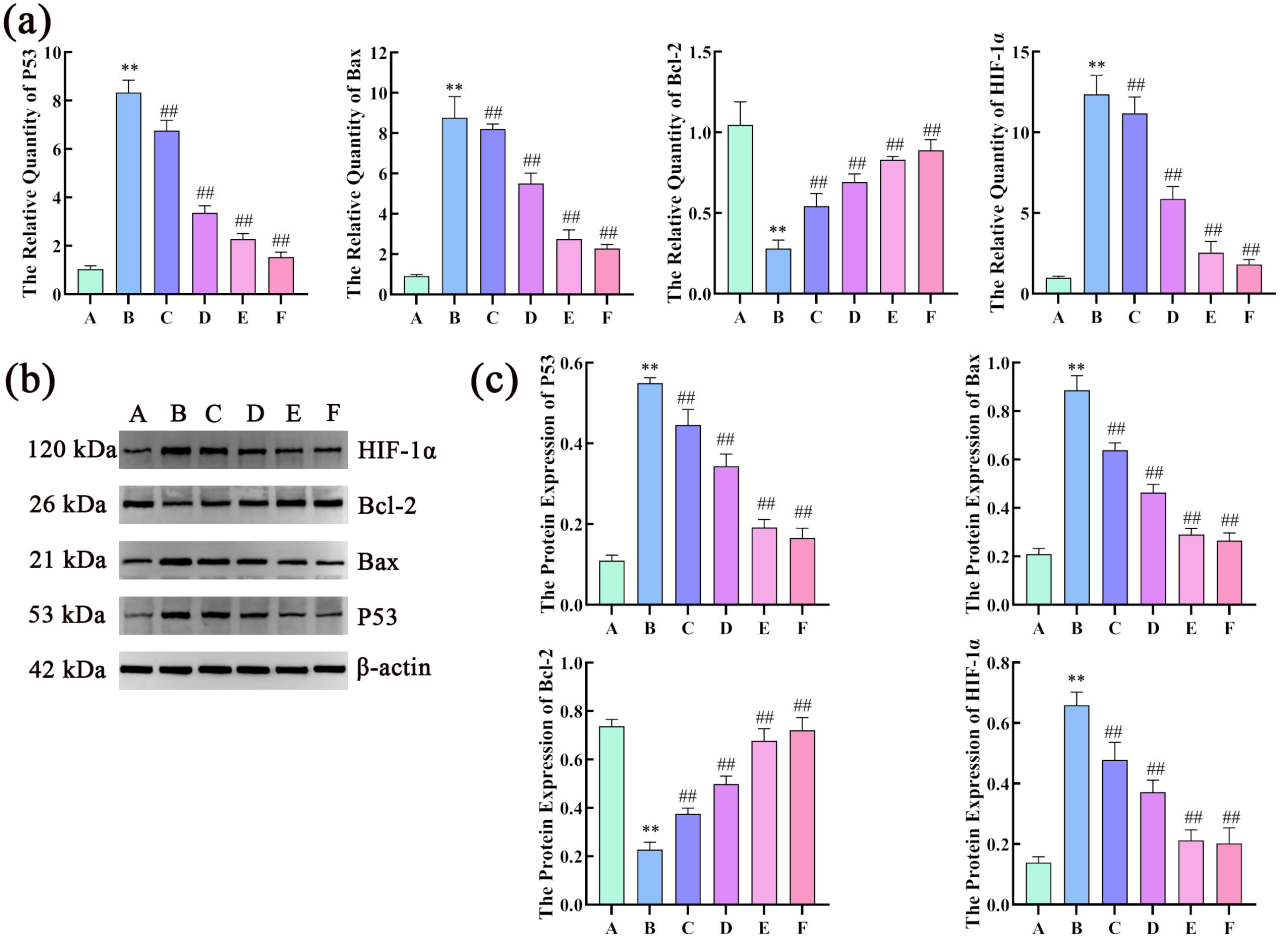
Effects of compound liver: protecting effervescent granules on P53, Bax, Bcl-2, and HIF-1α mRNA and protein expression levels. **(a)** Effects of CEHG on the expression of P53, Bax, Bcl-2, and HIF-1 mRNA α in rats with liver injury (*n* = 6, compared with the control group, **P < 0.01, compared with the model group, ^##^*P* < 0.01); **(b)** Gel electrophoresis images of P53, Bax, Bcl-2, and HIF-1α; **(c)** Quantification of gel electrophoresis of P53, Bax, Bcl-2, and HIF-1α Proteins (*n* = 3, compared with the control group, **P < 0.01, compared with the model group, ** *P* < 0.01).

The WB results showed that, compared with the control group, the model group had significantly higher expression of P53, Bax and HIF-1α protein, and decreased expression of protein Bcl-2. Compared with the model group, the dosed groups had significantly lower protein levels of P53, Bax, and HIF-1α protein levels but higher Bcl-2 levels (Figure 9).

## 4 Discussion

In this study, CEHG were developed using G*anoderma lucidum, Paeonia lactiflora*, and *Glycyrrhiza glabra* as raw materials, with the aim of exploring their auxiliary protective effect against CLI. Based on TCM theory and modern pharmacological research, a multi-dimensional study was conducted to systematically optimize the preparation process and clarify the hepatoprotective mechanisms involved, providing a scientific basis for the development of functional hepatoprotective foods.

In the optimization of the extraction process, orthogonal experiments, characterized by advantages of reducing the number of experiments required, thus saving resources and time, combined with the determination of the contents of hepatoprotective active components via HPLC, laid the foundation for process screening. The AHP-entropy weight method (28), by integrating the subjective weights of AHP and the objective weights of the entropy method, balanced subjective and objective factors. The combination of the dry extract rate and active hepatoprotective components was used as the characterization index for the entire formula, and the generalized score was applied to more accurately reflect the extraction quality. Eventually, an optimal extraction process was determined as a material-to-liquid ratio of 1:20, with 1 extraction per 1 h. Verification experiments showed that this process was stable, feasible, and reproducible.

The Box–Behnken response surface method was adopted to optimize the granulation process (29). This efficient and flexible three-level experimental design, suitable for quadratic response surface models, enabled sufficient information to be obtained with fewer experiments. The results indicated that the interaction between ethanol volume fraction and binder dosage had a significant impact on granule formation. The optimized granulation parameters (11 parts of diluent, 12% binder, and 80% ethanol volume fraction) were verified to meet the requirements of the Chinese Pharmacopeia, suggesting that in actual production, strict control of the ethanol volume fraction and binder dosage was necessary to avoid quality fluctuations.

Network pharmacology studies provided theoretical clues for the hepatoprotective mechanism of the CEHG: 121 active components were subjected to target prediction and intersection analyses with liver injury disease targets, resulting in 199 overlapping targets involving key signaling pathways such as HIF-1 (30), p53 (31), and FoxO (32). Further analysis of the active component-target-disease network and PPI network identified core components including palmitic acid ergosta-7,22-diene-3β-ol ester and paeoniflorin, as well as key targets such as CTNNB1 and P53, suggesting that the granules may exert their effects through multiple components, multiple targets, and multiple pathways.

Animal experiments were used to verify the aforementioned mechanisms. In the CCl4-induced liver injury model, the granules dose-dependently reduced serum liver function indicators such as AST and ALT and improved pathological damage to liver tissue, indicating their ability to alleviate liver injury. Meanwhile, the granules significantly reduced serum levels of inflammatory factors, including IL-6, IL-1β, and TNF-α, suggesting that they exert protective effects by inhibiting inflammatory responses. In terms of oxidative stress (33), the granules increased the activities of SOD and GSH-Px, as well as the content of GSH in liver tissue, and reduced the levels of MDA and ROS, indicating their capacity to enhance antioxidant capacity and alleviate damage from oxidative stress. Cell apoptosis detection revealed that granules could reduce the proportion of apoptotic cells in liver tissue, down-regulate P53 and Bax mRNA and protein expression, and up-regulate Bcl-2 expression, suggesting that they inhibit excessive hepatocyte apoptosis by regulating apoptosis-related molecules. The down-regulation of HIF-1α indicated that the granules might improve the hypoxic state of the liver, further alleviating oxidative stress.

In summary, this study provided reliable parameters for the large-scale production of CEHG granules through process optimization. Combined network pharmacology and animal experiments revealed that granules exert hepatoprotective effects through multiple pathways such as anti-inflammation, anti-oxidation, inhibition of apoptosis, and regulation of HIF-1α expression, offering solid scientific support for their further development and clinical application. In future studies, we plan to conduct more in-depth research on the underlying mechanism and increase the number of experimental replicates for the preparation process. This optimization is intended to improve the reproducibility and reliability of the experimental results. Additionally, we will add comparative experiments between effervescent granules and traditional dosage forms in terms of bioavailability, therapeutic efficacy, and other aspects to verify the advantages of effervescent granules.

## 5 Conclusion

This study determined the parameters for the extraction and molding process of CEHG, a hepatoprotective functional food. CEHG improved liver pathology, function indices, oxidative stress, and cell apoptosis of rats with CCl4-induced acute liver injury and regulated P53, Bax, Bcl-2, and HIF-1α expression to exert hepatoprotective effects.

## Data Availability

The raw data supporting the conclusions of this article will be made available by the authors, without undue reservation.
